# Physiological Function of Rac Prophage During Biofilm Formation and Regulation of Rac Excision in *Escherichia coli* K-12

**DOI:** 10.1038/srep16074

**Published:** 2015-11-04

**Authors:** Xiaoxiao Liu, Yangmei Li, Yunxue Guo, Zhenshun Zeng, Baiyuan Li, Thomas K. Wood, Xingsheng Cai, Xiaoxue Wang

**Affiliations:** 1 Key Laboratory of Tropical Marine Bio-resources and Ecology, Guangdong Key Laboratory of Marine Materia Medica, RNAM Center for Marine Microbiology, South China Sea Institute of Oceanology, Chinese Academy of Sciences, Guangzhou 510301, PR China; 2 University of Chinese Academy of Sciences, Beijing 100049, China; 3 Department of Chemical Engineering, Pennsylvania State University, University Park, Pennsylvania 16802-4400; 4 Department of Biochemistry and Molecular Biology, Pennsylvania State University, University Park, Pennsylvania 16802-4400.

## Abstract

Rac or rac-like prophage harbors many genes with important physiological functions, while it remains excision-proficient in several bacterial strains including *Escherichia coli*, *Salmonella* spp. and *Shigella* spp. Here, we found that rac excision is induced during biofilm formation, and the isogenic stain without rac is more motile and forms more biofilms in nutrient-rich medium at early stages in *E. coli* K-12. Additionally, the presence of rac genes increases cell lysis during biofilm development. In most *E. coli* strains, rac is integrated into the *ttcA* gene which encodes a tRNA-thioltransferase. Rac excision in *E. coli* K-12 leads to a functional change of TtcA, which results in reduced fitness in the presence of carbenicillin. Additionally, we demonstrate that YdaQ (renamed as XisR) is the excisionase of rac in *E. coli* K-12, and that rac excision is induced by the stationary sigma factor RpoS through inducing *xisR* expression. Taken together, our results reveal that upon rac integration, not only are new genes introduced into the host, but also there is a functional change in a host enzyme. Hence, rac excision is tightly regulated by host factors to control its stability in the host genome under different stress conditions.

Bacteriophages and bacteria are the most abundant life forms on Earth. Bacteria and phages also interact frequently, and each phage infection has the potential to introduce new genetic information into the bacterial host, thereby driving the evolution of bacteria. The introduction of novel genes by phages into the bacterial host can confer beneficial phenotypes that enable the exploitation of competitive environments^1^,^2^,^3^,^4^. Prophage-encoded virulence factors make important contributions to pathogenesis, including those of *Corynebacterium diphtheriae* (diphtheria), *Clostridium botulinum* (botulism), and *E. coli* O157:H7 (Shiga-like toxin)^5^. Active prophages such as Gifsy-2 can also give the *Salmonella* host a competitive advantage by killing competitors and by providing immunity^6^. In *E. coli*, the genes of cryptic prophage (i.e., prophage defective in plaque formation) provide multiple benefits to the host for survival in adverse environmental conditions, such as under oxidative, acid, osmotic, antibiotic stresses and biofilm formation^7^. Biofilm formation is arguably the dominant lifestyle for most bacteria, and prophage genes are among the most highly upregulated genes during biofilm development in *E. coli* and *Pseudomonas aeruginosa*^8^,^9^. In these cases, the reproductive success of the lysogenic bacterium carrying these new genes translates directly into an evolutionary success for the prophage resident in the chromosome.

In addition to contributing to the diversification of the bacterial genome architecture by introducing novel genes, another way for phages to affect the bacterial host is to disrupt or restore host genes upon prophage integration or excision. Prophage integration is commonly found in tRNA and tmRNA^10^. Integration into the coding region of genes has also been described, and the prophage attachment site frequently complements the coding sequence of the protein^11^. In some cases, prophage integration or excision does not disrupt the gene serving as the attachment site. For example, the Gifsy-1 prophage of *Salmonella* integrates within the coding sequence of *lepA* and does not change *lepA* function^12^. Similarly, prophage e14 in *E. coli* K-12 inserts within the isocitrate dehydrogenase gene^13^, and Φ297 in *E. coli* O157:H7 integrates at the *yecE* site^14^ without changing the gene function. However, in other cases, inactivation of protein function by prophage integration or excision has also been described. For example, a lipase gene undergoes negative lysogenic conversion by a *Staphylococcus aureus* phage^15^,^16^. In the human pathogen *Listeria monocytogenes,* integration of prophage A118 disrupts the host gene *comK* encoding the major competence transcription factor^17^, and excision of A118 restores the function of *comK*^18^.

Prophage rac was the first defective prophage discovered in *E. coli* K-12^19^,^20^, and it has been regarded as a phage fossil that was acquired over 4.5 million years ago^21^. The rac prophage in the laboratory *E. coli* strain K-12 and two rac-like prophages (Sp10 and CP-933R) in two strains of enterohemorrhagic *E. coli* O157:H7 (Sakai and EDL933) all integrate into the *ttcA* gene, which encodes a tRNA-thioltransferase in these three genomes^22^,^23^. The leftmost 21 kb are greater than 99.9% identical in Sp10 and CP-933R, and the leftmost 8 kb are 99.0% identical to the K-12 rac prophage^23^. Rac harbors 27 genes and 5 pseudogenes in *E. coli* K-12, and deletion of the entire rac prophage reduces resistance to acid stress, oxidative stress and antibiotic stress^7^. The prophage proteins in rac responsible for inhibiting cell division, including KilR, appear important for resistance to nalidixic acid and azlocillin^7^,^24^. YdaC in rac was identified in a screen for antibiotic resistance using pooled plasmids from the ASKA library that showed increased resistance to erythromycin^25^. The type I toxin-antitoxin pair RalR-RalA in rac increases resistance to fosfomycin^26^. In addition, RecE and RecT in rac are involved with RecA-dependent recombination^27^. Although it harbors important genes, rac remains excision proficient in *E. coli* K-12 and in the Sakai strain^7^,^22^. In *E. coli* K-12, rac has the second highest excision rate under normal growth conditions. Excision of the rac prophage would necessarily lead to the loss of rac genes due to the lack of capacity to replicate once excised^28^. Although rac and Sp10 are both lambda-like prophages, unlike lambda prophages, their excision is not induced upon mitomycin C treatment, which is known to trigger the host SOS responses^22^,^29^. Hence, two major questions remain for rac prophage excision: whether rac excises under stressed conditions and whether rac excision affects host gene function. In this study, we addressed both questions and found that rac excision in *E. coli* K-12 is induced during biofilm formation, and we found that the rac deletion strain formed more biofilm in nutrient rich medium at early stages and showed high swimming motility. The function of the host gene *ttcA* was assessed before and after rac excision under carbenicillin stress, and the results showed that rac integration caused a functional change in TtcA by creating a variant of the protein while rac excision restored it to the ancient copy. In addition, we identified the excisionase gene for rac and propose to name it *xisR* (excisionase gene for rac). XisR induced rac excision and bound to the rac attachment site. Furthermore, in addition to the previously identified host factor H-NS, we found that the sigma factor RpoS also increased rac excision by regulating the transcription of *xisR* at the stationary phase.

## Results

### Rac excision is induced during biofilm formation

**Figure 1:**
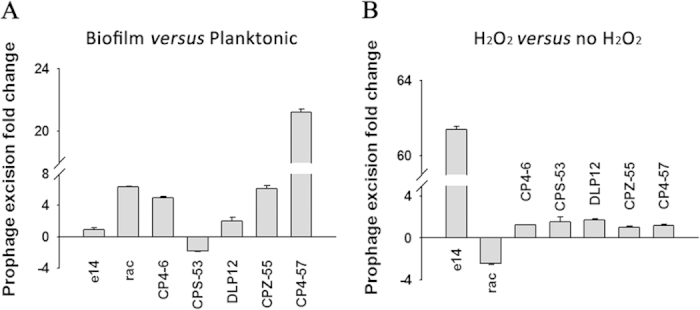
Fold change of the frequencies of prophage excision in *E. coli* K-12. (**A**) Fold change of excision of seven prophages in biofilm cells *versus* planktonic cells at 48 h in *E. coli* K-12. (**B**) Fold change of excision of seven prophages with oxidative stress (2 mM H_2_O_2_) *versus* without oxidative stress in *E. coli* K-12. H_2_O_2_ was added to cells (OD_600_ ~ 1.0) for 90 min. Data are from two independent cultures and one standard deviation is shown in A and B.

Biofilms are the preferred lifestyle of bacteria, and prophages are involved in biofilm formation^8^,^30^; therefore, we tested whether prophage excision is regulated during biofilm formation in *E. coli* K-12 BW25113. To test the frequency of excision of the nine prophages, the fraction of cells that undergoes excision was quantified by quantitative PCR (qPCR) in a total of ~10^9^ cells using the method described previously^7^. When compared to planktonic cells, the frequency of rac excision increased 6.4 ± 0.1-fold (3 per 10^5^ cells *vs* 0.5 per 10^5^ cells) in 48 h biofilm cells, and the frequencies of excision of cryptic prophages CP4-6 (1.5 per 10^5^ cells *vs* 0.3 per 10^5^ cells) and CPZ-55 (0.5 per 10^6^ cells *vs* 0.8 per 10^7^ cells) were also increased but with lower overall excision (Fig. 1A). Similar to what has been reported previously, the frequency of CP4-57 excision increased 21.2 ± 0.2-fold in the biofilm cells (1.6 per 10^6^ cells) when compared to the planktonic cells (7.5 per 10^8^ cells)^8^. Prophage e14 had the highest frequency of excision (5.5 per 10^4^ cells) in BW25113; however, e14 excision was not induced in biofilm cells (Fig. 1A). Excision of prophage Qin or CP4-44 was not detected (<1 per 10^8^ cells), which is likely due to the lack of an active integrase for Qin and CP4-44^7^,^31^. As oxidative stress is involved in biofilm formation, we tested whether oxidative stress (using 2 mM hydrogen peroxide) affected prophage excision in BW25113 cells^32^. Prophage e14 was the only prophage induced to excise with the addition of hydrogen peroxide (61.4 ± 0.1-fold), and rac was the only prophage repressed with the addition of hydrogen peroxide (1.9  ± 0.1-fold) (Fig. 1B). Taken together, these results show that rac excision is induced in biofilm cells but not under oxidative stress condition.

### Rac genes affect biofilm formation and motility

**Figure 2:**
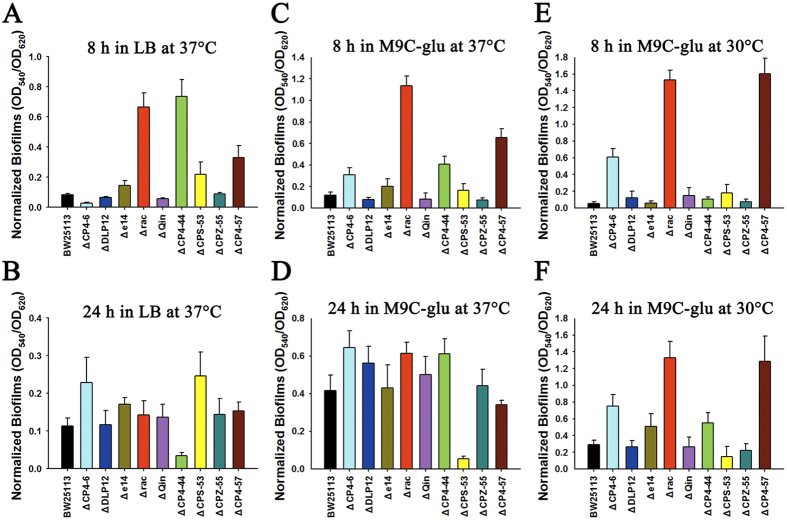
Biofilm formation in nine prophages deletion strains. Early (**A**,**C**,**E**) and late (**B**,**D**,**F**) biofilm formation of *E. coli* K-12 BW25113 and nine isogenic prophage deletion mutants were tested in two different media (LB or M9C-glu) at two different temperatures (37 °C or 30 °C), respectively. Each data point is the average of at least ten replicate wells from two independent cultures, and one standard deviation is shown here from (**A–F**).

As rac is excised during biofilm formation, we tested how rac genes affect biofilm formation using the previously obtained prophage deletion strain Δrac^28^, constructed by us through overexpressing an engineered *hns* which specifically induces rac excision. When tested in rich LB medium at 37 °C, the Δrac strain formed 7.9 ± 0.2-fold more biofilm compared to the BW25113 wild-type cells at an 8 h early stage and showed the second highest fold change among the nine prophage deletion strains (Fig. 2A). However, when measured at 24 h, the biofilm formed by Δrac strain was comparable to the wild-type strain (1.3 ± 0.3-fold) (Fig. 2B). Additionally, when using M9C minimal medium supplemented with 0.4% glucose (M9C-glu) at 37 °C, the Δrac strain formed the largest amount of biofilm among the nine prophage deletion strains compared to the wild-type strain (9.5 ± 0.8-fold) at 8 h (Fig. 2C) but was comparable to the wild-type at 24 h (1.5 ± 0.2-fold) (Fig. 2D). In our previous study, we found that the Δrac strain in M9 casamino acids (M9C) medium and at 30 °C, as well as seven of the prophage deletion strains (except for ΔCP4-57 strain), formed less biofilm than the wild-type strain at 8 h^7^. To further test whether biofilm formation in the Δrac strain is nutrient-dependent, we tested biofilm formation at the same temperature but with the addition of 0.4% glucose (M9C-glu). Consistent with the results in LB and M9C-glu at 37 °C, the Δrac strain formed more biofilm than the wild-type, with a 27.9 ± 0.3-fold increase at 8 h (Fig. 2E) and a 4.6 ± 0.3-fold increase at 24 h (Fig. 2F). Prophage CP4-57 also undergoes induced excision during biofilm formation; we found that the ΔCP4-57 strain formed more biofilm in M9C-glu medium (Fig. 2E,F).

**Figure 3:**
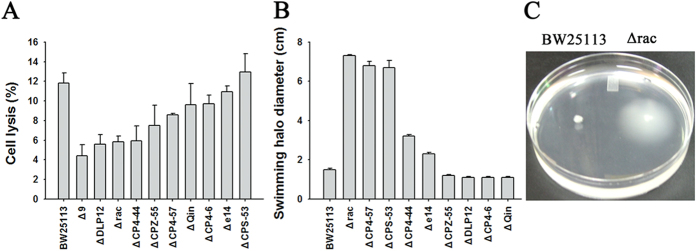
Rac genes affect cells lysis and cell motility. (**A**) Cell lysis in nine prophage deletion strains as measured in 48 h biofilm cells. (**B**) Motility of nine prophage deletion strains in low salt motility agar plates. Data are from two independent cultures and one standard deviation is shown in A and B. (**C**) Motility of Δrac and wild-type strains on high salt motility plates. Photographic image was taken by one of the first authors XL. All of the isogenic strains constructed did not contain any insertion elements in the promoter region of *flhDC* operon.

As prophage excision has been associated with cell lysis during biofilm formation^33^, we assessed whether prophage genes contribute to cell lysis by comparing lysis in the prophage deletion strains to the BW25113 strain. Among the nine prophages, deletion of rac and DLP12 resulted in the most significant reduction in cell lysis with 5.8% ± 0.6% and 5.6% ± 0.9% of the cells lysed, respectively, compared to 11.8% ± 1.0% of the cells lysed in the wild-type biofilm cells (Fig. 3A). Furthermore, we tested the change in swimming motility in the Δrac strain. As shown in Fig. 3B,C, the Δrac strain showed much higher motility than the wild-type strain (7.3 ± 0.1-fold). Additionally, ΔCP4-57 and ΔCPS-53 strains also showed much higher motility than the wild-type strain (Fig. 3B). We and other groups have reported that the increased motility of BW25113 isogenic strains could also be caused by transposition of insertion elements (e.g., IS*5* or IS*1*) into the master flagella operon *flhDC*^34^,^35^, which leads to a high and constitutive expression of flagella genes. Therefore, all of the prophage deletion strains used here were screened for the presence of insertion elements in *flhDC* by PCR and qPCR, and we verified that all of the isogenic strains constructed did not contain any insertion elements in *flhDC.*

### Rac excision causes amino acid changes in TtcA

**Figure 4:**
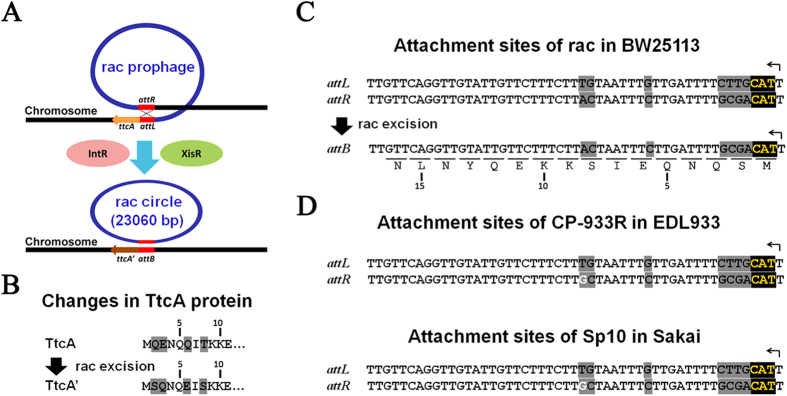
Changes in TtcA before and after rac excision. (**A**) Excision of rac was induced by overproduction of excisionase and integrase proteins in *E. coli* K-12 BW25113. (**B**) Rac excision caused a change of four amino acids of TtcA at the N-terminus. Numbers on the top indicate the amino acid position in TtcA. (**C**) The nucleotide sequence of the left and the right attachments of rac before excision (*attL* and *attR*) as well as the attachment site after rac excision (*attB*) in *E. coli* K-12 BW25113. Letters labeled under the *attB* sequence indicate the partial TtcA’ amino acid sequence. Numbers on the bottom indicate the amino acid position in TtcA’. (**D**) The nucleotide sequence of the two attachments sites (*attL* and *attR*) of rac-like prophage, CP-933R in *E. coli* O157:H7 Str. EDL933 strain and Sp10 in *E. coli* O157:H7 Str. Sakai strain. Arrows indicate the translational start of TtcA.

The prophage rac resides between *ttcA* and the pseudogene *ttcC*’ in *E. coli* K-12 BW25113 (Fig. 4A). By comparing the DNA sequences near the attachment region before rac excision (in the wild-type strain) and after excision (in the Δrac strain), we found that excision of rac results in a change of four TtcA amino acids near the N-terminus (Fig. 4B) due to the imperfect match of the left and the right attachment sites (Fig. 4C). The same change was obtained when the rac excisionase is overproduced, which confirms that this change is indeed caused by the natural excision of rac. Two previously identified rac-like prophages, CP-933R and SP10, are both inserted into the same *ttcA* site of *E. coli* O157:H7 Str. EDL933 and *E. coli* O157:H7 Str. Sakai, respectively^23^. Through a bioinformatics search, we found that although there are differences (one nucleotide) among the right attachment sites of these three strains, the same changes in the TtcA protein should occur when the rac and rac-like prophages are excised from the host genome of these two strains (Fig. 4D).

**Figure 5:**
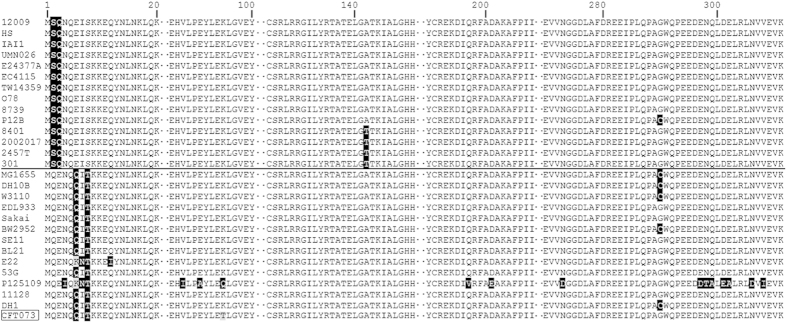
Sequence alignment of TtcA in bacteria without or with rac integrated. Alignment of TtcA sequences was performed using the ClustalX program. Sequences from 14 strains without rac integrated at *ttcA* (above the black line) and from 13 bacteria strains with rac integrated at *ttcA* (below the black line) as well as the *E. coli* CFT073 strain (box indicated) with rac integrated elsewhere other than *ttcA* gene. Numbers on the top indicate the amino acid position in TtcA. Portion with identical amino acids in the 28 species are excluded (dot indicated), and only non-identical amino acids are highlighted. Species abbreviations: 12009, *E. coli* O103:H2 Str.12009; HS, *E. coli* HS; IAI1, *E. coli* IAI1; UMN026, *E. coli* UMN026; E24377A, *E. coli* E24377A; EC4115, *E. coli* EC4115; TW14359, *E. coli* O157:H7 Str. TW14359; O78, *E. coli* APEC O78; 8739, *E. coli* ATCC 8739; P12B, *E. coli* P12B; 8401, *Shigella flexneri* 5 Str. 8401; 2002017, *Shigella flexneri* 2002017; 2457T, *Shigella flexneri* 2A Str. 2457T; 301, *Shigella flexneri* 2A Str. 301, MG1655, *E. coli* K-12 MG1655; DH10B, *E. coli* K-12 DH10B; W3110, *E. coli* K-12 W3110; EDL933, *E. coli* O157:H7 Str. EDL933; Sakai, *E. coli* O157:H7 Str. Sakai; BW2952, *E. coli* BW2952; SE11, *E. coli* SE11; BL21, *E. coli* BL21 (DE3); E22, *E. coli* E22; 53G, *Shigella sonnei* 53G; P125109, *Salmonella enterica* subsp. serovar Enteritidis Str. P125109; 11128, *E. coli* O111:H- Str. 11128; DH1, *E. coli* DH1; CFT073, *E. coli* CFT073.

To explore the possibility that rac affects *ttcA* upon its integration, we analyzed the TtcA sequences and the upstream and downstream regions of *ttcA* in all of the *E. coli* strains available in the EcoGene-RefSeq database (http://www.ecogene.org/refseq). We also included closely related strains of *Shigella* and *Salmonella* that harbor rac or rac-like prophages in our analysis. The alignment results show that although the N-terminus of TtcA is rather variable, the protein can be categorized into two distinctive groups depending on whether or not they harbor rac-like prophages (Fig. 5). For the 10 strains of *E. coli* and 4 strains of *Shigella flexneri* without a rac-like prophage inserted in *ttcA,* such as *E. coli* O103:H2 Str. 12009, *E. coli* UMN026, and *Shigella flexneri* 5 Str. 8401 (Fig. 5, above black line), the N-terminus of TtcA is the same as the TtcA’ variant created by rac excision in *E. coli* K-12 strains. While in the other 13 strains harboring rac or rac-like prophages in *ttcA*, such as *E. coli* E22, *Shigella sonnei* 53G, and *Salmonella enterica* subsp. serovar Enteritidis Str. P125109, the N-terminus of TtcA is the same as the *E. coli* K-12 strain with rac (Fig. 5, below black line). These results suggest that the TtcA’ variant formed after rac excision in *E. coli* K-12 is the ancient copy of this protein and that rac integration changed the ancient TtcA. One exception is the uropathogenic *E. coli* strain CFT073, which does not harbor a rac-like prophage in *ttcA* but does possess a Φ-CTF073-*potB* prophage inserted into the *potB* gene. Prophage Φ-CTF073-*potB* carries similar structural genes (e.g., capsid and tail protein) and a recombination gene (e.g., *recE*) to those found in rac-like prophages^36^.

### Rac excision causes a functional change in TtcA

**Figure 6:**
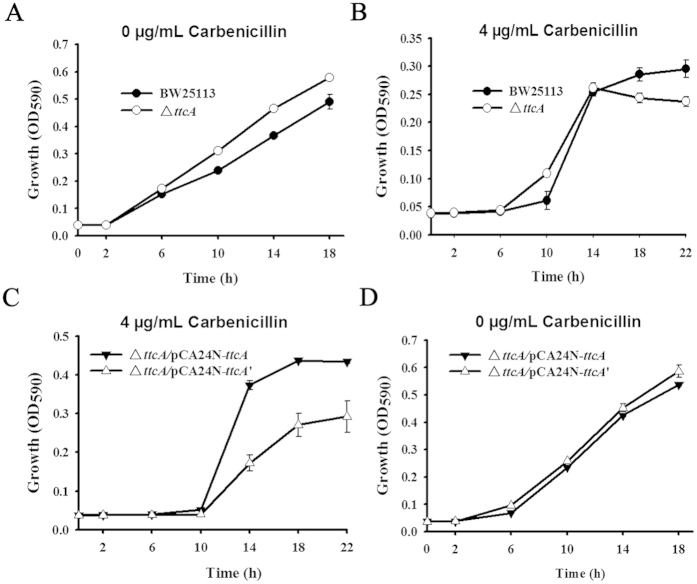
Rac excision causes a functional change in TtcA. Metabolic rate of the wild-type and Δ*ttcA* strains in the absence of carbenicillin (**A**) or in the presence of 4 μg/mL carbenicillin (**B**). Metabolic rate of the Δ*ttcA* strain overexpressing a wild-type TtcA (before rac excision) or overexpressing TtcA’ (TtcA variant after rac excision) with 4 μg/mL carbenicillin (**C**) and in the absence of carbenicillin (**D**). Data are from two independent cultures and one standard deviation is shown.

Large-scale phenotypic screening of all Keio mutants from PortEco showed that the Δ*ttcA* knockout strain has an altered fitness score when exposed to carbenicillin^37^. As shown in Fig. 6A, the Δ*ttcA* strain showed increased metabolic activity compared to that of the wild-type strain without carbenicillin. In contrast, in the presence of 4 μg/mL carbenicillin, the Δ*ttcA* strain showed reduced metabolic activity compared to that of the wild-type strain at later times (after 14 h) (Fig. 6B). Since the rac left attachment site (*attL*) is inside the coding region of *ttcA*, in the Δ*ttcA* strain, the removal of the whole *ttcA* coding region would completely abolish rac excision. In order to investigate whether rac excision causes a functional change in TtcA, a native TtcA (before rac is excised) and the TtcA’ variant (after rac is excised) were produced in the same Δ*ttcA* host strain, thereby ruling out the potential effect caused by differences in the frequency of rac excision in the wild-type strain and in the Δ*ttcA* strain. The results show that the cells producing native TtcA have a much higher metabolic rate than the TtcA’ variant in the presence of carbenicillin, which suggests that rac excision causes a reduction in the ability of TtcA to cope with carbenicillin stress (Fig. 6C). As a negative control, the metabolic rate of cells overexpressing the native TtcA was comparable to cells overexpressing TtcA’ in the absence of carbenicillin (Fig. 6D). Taken together, these results show that rac excision causes a change in the host gene *ttcA* that leads to reduced fitness in the presence of carbenicillin stress.

### YdaQ is the rac excisionase

**Figure 7:**
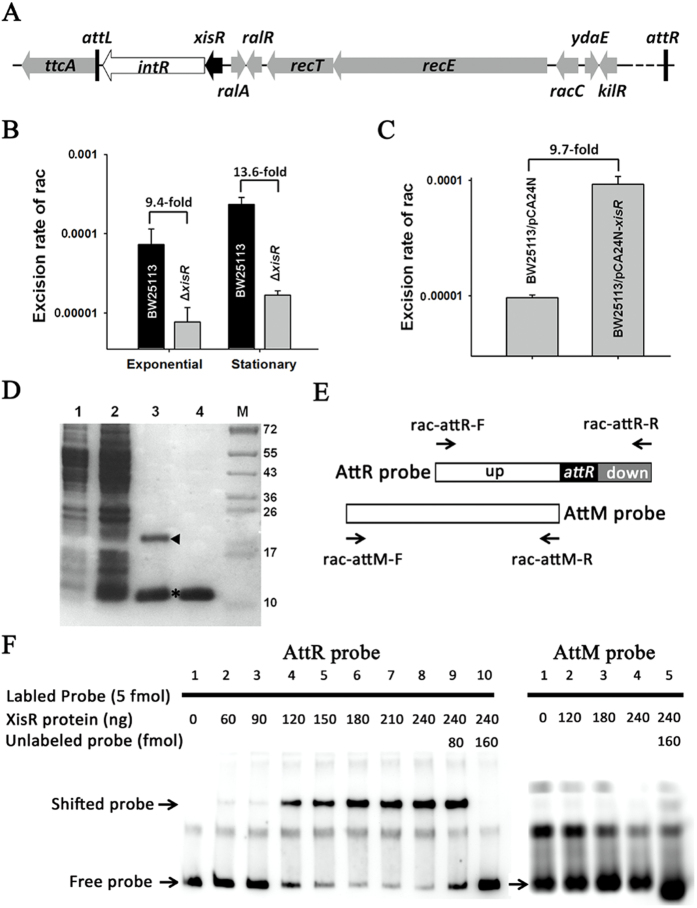
YdaQ (XisR) controls rac excision. (**A**) Schematic representation of the *intR* and *xisR* genes as well as the neighboring genes of prophage rac. (**B**) Excision rates of rac in the Δ*xisR* and in BW25113 strains. (**C**) Excision rates of rac in the wild-type strain overexpressing *xisR* via pCA24N-*xisR*. IPTG (1 mM) was added to BW25113/pCA24N-*xisR* cells (OD_600_ ~ 1.0) for 4 h. Error bars represent standard deviations for triplicate cultures in B and C. (**D**) XisR was purified via pET28b-*xisR*. IPTG (1 mM) was added to induce *xisR* expression for 4 h (lane 2), and cells without IPTG were included as a negative control (lane 1). Possible dimers of XisR were observed during purification (lane 3), and the addition of the reducing agent dithiothreitol (DTT) reduced the dimerization of XisR (lane 4). The triangle indicates the dimers of XisR, and the asterisk indicates the XisR monomers. (**E**) Schematic representation of the PCR amplified fragments, AttR (294 bp) and AttM (291 bp), which are used as probes in EMSA. The AttR fragment contains the rac *attR* region and *attR* upstream and downstream regions, while AttM only contains the upstream region of *attR*. (**F**) EMSA results show that the purified XisR binds to the biotin-labeled AttR fragment and that, the binding increases with increasing concentrations of XisR. As a negative control, XisR does not bind to the biotin-labeled AttM fragment. Equal amounts of labeled probe was added in each lane.

Excisionase enzymes are required for lambdoid prophage excision by interacting with integrase^38^, and the integrase gene is usually adjacent to the attachment site on the chromosome in order to interact with its DNA target^39^. In rac, an integrase *intR* is found next to the *ttcA* gene in which the 5′-end serves as the left attachment site in *E. coli* K-12^23^. Next to *intR* lies *ydaQ*, which encodes a small protein of 71 amino acids (Fig. 7A). Here, we tested the ability of YdaQ to induce rac excision. When *yadQ* is deleted, rac excision was reduced 9.4 ± 0.8-fold during the exponential phase and 13.6 ± 0.3-fold during the stationary phase (Fig. 7B), while overexpressing *ydaQ* increases rac excision 9.7 ± 0.2-fold (Fig. 7C). Deletion of other genes nearby *intR*, such as *ralR* and *ralA*, did not affect rac excision (data not shown). To determine whether YadQ can bind the attachment site like other excisionases, we performed electrophoresis mobility shift assays (EMSA) using purified YdaQ with an N-terminal hexahistidine tag (His-tagged) (Fig. 7D) which is verified by mass spectrometry (Supplementary Table S1). Two DNA fragments, one containing the right attachment site of rac (AttR), and one that does not contain the right attachment site of rac (AttM) were used as probes (Fig. 7E). The EMSA results show that YdaQ bound and shifted the right attachment site of rac. In addition, the binding increased with the increasing concentrations of YdaQ (Fig. 7F, AttR panel). As a negative control, YadQ did not bind to the biotin-labeled AttM fragment (Fig. 7F, AttM panel). In addition, possible dimers were observed when YadQ was purified (Fig. 7D, lane 3), and the addition of the reducing agent dithiothreitol (DTT) greatly reduced the dimerization of YdaQ (Fig. 7D, lane 4). Taken together, these results suggest that YdaQ controls rac excision, and thus, we propose to rename *ydaQ* as *xisR* (excisionase for rac prophage).

### Rac excision is regulated by RpoS through excisionase

**Figure 8:**
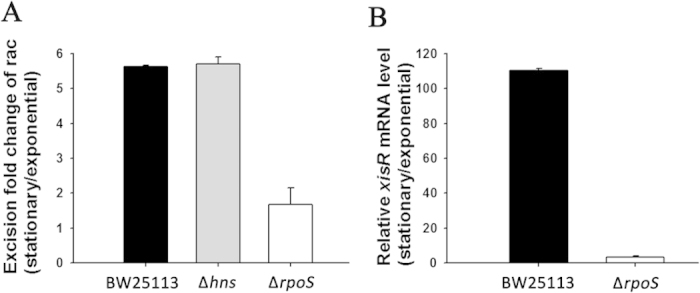
RpoS regulates rac excision by inducing *xisR* expression. (A) Excision rates of rac in BW25113, Δ*hns*, and Δ*rpoS* strains were measured during two different growth phases. Fold change indicates the change of rac excision rates in the stationary phase *versus* in the exponential phase. (B) Transcripts of *xisR* in the in the BW25113 and in Δ*rpoS* strains at two different growth phases. Fold change indicates the change of gene expression in the stationary phase *versus* in the exponential phase. Expression of *rrsG* was used to normalize the total RNA in different samples. Data are from two independent cultures and one standard deviation is shown in A and B.

We found that rac has a higher excision rate in stationary phase growth compared to exponential phase growth (Fig. 7B). Our previous results showed that rac excision was regulated by the host factor H-NS during the stationary phase^28^. In this study, we found that cells lacking H-NS still exhibit a higher excision rate in the stationary phase compared to exponential phase (5.7 ± 0.2-fold) (Fig. 8A), which suggests that other host factors besides H-NS may also control rac excision. We next tested whether rac excision is regulated by the stationary phase sigma factor RpoS. After deleting *rpoS*, there was no significant difference in rac excision between the cells in the exponential growth phase and in the stationary growth phase (1.7 ± 0.5-fold) (Fig. 8A), which suggests that RpoS is involved with rac excision. The qRT-PCR results show that the expression level of *xisR* was highly induced (110.3 ± 1.0-fold) in the wild-type strain but was only slightly induced in the *rpoS* deletion strain from the exponential phase to stationary phase (3.4 ± 0.5-fold) (Fig. 8B). Taken together, these results show that rac excision is induced in the stationary phase and that RpoS plays a role in regulating *xisR* expression.

## Discussion

Collectively, our results support the hypothesis that rac integration or excision affects host function, especially under stress conditions. In this study, we tested whether rac integration or excision affects the function of the protein encoded by the host gene *ttcA,* which serves as one of the attachment sites for rac in *E. coli* K-12. We found that the amino acid sequence and function of TtcA were altered after rac excised. Previously, we have shown that the excision of rac decreased cell survival in the presence of nalidixic acid and azlocillin primarily through the KilR protein, which inhibits cell division^7^,^40^. Recently, we also found that the deletion of the type I toxin/antitoxin pair RalR/RalA in rac reduced metabolic activity in the presence of fosfomycin^26^. Conversely, studies have also shown that deletion of the complete rac prophage greatly increased resistance to bicyclomycin, which functions through inhibition of the termination factor Rho, and the resistance is conferred by deletion of *kilR* and the remaining downstream operon but not DNA downstream of *kilR*^41^. Taken together, these results reveal that the integration or excision of the rac prophage can lead to a change in host fitness in the presence of different antibiotics. Thus, prophages can increase host fitness in the presence of antibiotics not only by introducing new genes (e.g., *kilR*, *ralRA*) into the host but also by affecting the function of the host protein by altering the coding sequence of the protein upon integration.

In this study, we found that rac excision is induced during biofilm formation (Fig. 1A), which is supported by the increased excision seen in the stationary phase (Fig. 7B), as biofilm cells are most similar to stationary phase cells^42^. Two proteins from rac, KilR and RalR, can both lead to cell death when overproduced^24^,^26^, but whether RalR or KilR can trigger cell death or cell lysis in biofilms requires further investigation. Conversely, recent studies have also shown that prophage excision under a specific condition, such as biofilm formation, will create genetic or potential phenotypic variation at the population level by generating a sub-population of prophage-free cells, which may benefit the population as a whole^43^,^44^. Here, we found that the rac deletion strain showed high swimming motility and formed more biofilm at an early stage in nutrient-rich medium. Excision of prophage CP4-57 in *E. coli* K-12 is induced during biofilm formation at early stages in both nutrient-rich and nutrient-poor medium, which led to a formation of slow-growing cells with increased motility^8^. In *Pseudomonas aeruginosa*, the conversion of prophage Pf4 into the super infective form occurred in dispersed biofilms, which is correlated with the appearance of small colony variants in the dispersal population^44^. In *Streptococcus pneumoniae*, a small population of cells undergoes prophage induction and contributes to the eDNA release during biofilm formation^45^. The excision of prophage A118 is specifically induced during intracellular growth primarily within phagosomes, which restores a functional competence gene that is required for the efficient phagosomal escape of *L. monocytogenes*^18^. These results suggest that bacteria can use various ways to respond to stress that involve editing their genomes through prophage excision. Hence, prophages not only contribute to the diversification of genome architecture among different bacterial strains but also contribute to intra-population diversification in a biofilm or in association with a eukaryotic host.

Previous attempts to identify prophage excision under normal growth conditions have shown very low excision frequencies^7^,^46^,^47^, which indicates stable residence of prophages under these conditions. To date, several host proteins have been described to control the expression of foreign DNA in bacteria. H-NS plays a key role in selectively silencing the transcription of horizontally acquired genes in Gram-negative bacteria^48^,^49^,^50^. In the nine prophages in *E. coli* K-12, H-NS only controls the excision of rac, which suggests a selective repression of H-NS on different prophages^28^. Similarly, the DNA-binding protein Hha only induces cryptic prophage CP4-57 and DLP12 excision^8^, and the transcription terminator factor Rho only represses cryptic prophage CPS-53 excision^51^. For the prophages CP4-57 and DLP12, Hha was found to bind to the attachment sites of these two prophages via nickel-enrichment DNA microarrays, thus facilitating homologous recombination during prophage excision^52^. In addition, the alternative sigma factor RpoH (σ^H^) in *Staphylococcus aureus* is recruited by a temperate phage to regulate prophage integration and excision^46^. Here, we found that the stationary sigma factor RpoS regulates rac excision. Taken together, these results collectively reveal that recruitment of host factors by phages for integration/excision modulation may provide a novel strategy to sense host conditions and further influence prophage fitness and the correlative bacterial fitness. In addition, unlike rac in *E. coli* K-12, two rac-like prophages in *E. coli* O157:H7 retain replication machinery and can further infect other strains, including *E. coli* K-12, once excised^20^. Therefore, further study on the excision and transmissibility of rac or rac-like prophages under different stress conditions in other pathogenic strains is necessary.

## Methods

### Bacterial strains, plasmids, and growth conditions

The bacterial strains and plasmids used in this study are listed in Table 1. The experiments were conducted in Luria-Bertani (LB) medium^53^ at 37 °C unless specified. Kanamycin (50 μg/mL) was used for culturing strains with single-gene knockouts from the Keio Collection^54^, chloramphenicol (30 μg/mL) was used for maintaining the pCA24N-based plasmids, and kanamycin (50 μg/mL) was used to maintain the pET28b-based plasmids.

### Plasmid construction

The primers used for the construction of the plasmids are listed in Table S2. For the construction of pCA24N-*ttcA* and pCA24N-*ttcA*’, the coding region of the target gene was PCR amplified from genomic DNA isolated from the BW25113 and from the Δrac strains, respectively. The PCR products were phosphorylated, purified, and ligated into vector pCA24N as previously described^26^,^55^. The ligation mixture was transformed into BW25113 strains. The constructs were confirmed by PCR followed by DNA sequencing using primers pCA24N-F and pCA24N-R. For the construction of pET28b-*xisR*, the coding region of *xisR* was PCR amplified using the primer pair pET28b-*xisR*-F/R, the PCR product was digested with *Nco*I and *Hind*III, and the digested PCR product was ligated into digested pET28b. The construct was confirmed by PCR followed by DNA sequencing using primers T7-F and T7-R.

### Biofilm cell collection

Biofilm cells were collected as previously described with minor modifications^56^. Overnight cultures of the BW25113 and isogenic prophage deletion strains were inoculated into a 2 L shake flask (to reach OD_600_ ~ 0.05) containing 250 mL LB and 10 g glass wool (Corning Glass Works, Corning, NY), during which time the cells formed a biofilm on the surface of the glass wool. After incubation with shaking at 180 rpm at 37 °C for 48 h, the planktonic cells in contact with biofilms were taken directly from the culture. At the same time, the glass wool was taken from the culture and quickly washed twice in 100 mL ice-cold 0.85% NaCl. The biofilm cells were removed from the glass wool by sonication at 22 Watts for 2 min in a FS3 water bath sonicator (Fisher Scientific, Pittsburgh, PA, USA) submerged in 200 mL of ice-cold 0.85% NaCl. The biofilm cells were collected by centrifugation at 4 °C for 30 s to remove the NaCl. The cell pellets from the planktonic state growth and biofilm growth were saved at -80 °C until used.

### Biofilm assay

Biofilm formation assays were carried out in 96-well polystyrene plates (Corning Costar, Cambridge, MA, USA) using 0.1% crystal violet staining^57^. Overnight *E. coli* K-12 BW25113 and the isogenic prophage deletion strain cultures were diluted to an OD_600_ of 0.05 in LB or M9C-glucose (M9 minimal medium with 0.4% casamino acids and 0.4% glucose) medium without shaking for 8 h or 24 h. The cell growth was measured at 620 nm. The biofilm cells were stained with 0.1% crystal violet and measured at 540 nm. To remove the growth effects, biofilm formation was normalized by bacterial growth for each strain. Two independent cultures were used for each strain.

### Biofilm lysis assay

As *E. coli* K-12 BW25113 and the isogenic prophage deletion mutants do not have a complete *lacZ* gene, the plasmid pLP170 was introduced to provide a constitutive expression of *lacZ*^58^,^59^. Thus, BW25113 cells harboring pLP170 cells will release *β*-galactosidase into the supernatant only when the cells are lysed. The biofilm cells were collected using the method described above with the exception that 100 μg/mL carbenicillin was added to maintain the pLP170 plasmid. After 48 h of incubation, the supernatant was collected after removing the cells in the culture, and the biofilm cells were collected after removal from the glass wool by sonication. The β-galactosidase activity was measured using the supernatant and the biofilm cells as described previously^58^. The percentage of lysis was calculated by quantifying the β-galactosidase activity in the supernatant compared to the total β-galactosidase activity in the biofilm cells and in the supernatant.

### Protein expression and purification

For protein purification, *E. coli* BL21 (DE3) cells containing plasmid pET28b-*xisR* were grown overnight in LB supplemented with kanamycin. The overnight cultures were diluted 1:100 in LB, and 1 mM IPTG was added when the OD_600_ reached ~1.0. The cells were collected by centrifugation after IPTG induction for 4 h, resuspended in lysis buffer (50 mM NaH_2_PO_4_·2H_2_O, pH 8.0, 300 mM NaCl) and were disrupted using a Fast-Prep 24 instrument (MP Biomedicals, Santa Ana, CA). The cell debris was removed by centrifugation, and the His-tagged protein was purified using Ni-NTA agarose (Qiagen) following the manufacturer’s instructions. The eluted His-tagged proteins were collected and dialyzed against buffer (20 mM Tris-HCl, 500 mM NaCl, pH 7.4) and were stored at 4 °C.

### Electrophoretic mobility shift assay (EMSA)

The DNA fragment AttR covering the *attR* site was PCR amplified using primer pair rac attR-F/R (Table S2). As a negative control, fragment AttM that only contains the upstream region of *attR* was also PCR amplified using primer pair rac-attM-F/R. DNA fragments were purified and labeled using the Biotin 3′ End DNA Labeling Kit (Thermo scientific, Rockford, USA). The purified protein and DNA fragments were mixed following the protocol as described in the LightShift Chemiluminescent EMSA kit (Thermo scientific, Rockford, USA). The binding reaction mixtures were incubated at 25 °C for 1 h. The protein-DNA complexes were separated by electrophoresis in 0.5 × Tris-borate-EDTA (TBE) polyacrylamide gels and were then transferred to nylon membranes. The DNA fragments were visualized using the Chemiluminescence Nucleic Acid Detection Module Kit (Thermo scientific, Rockford, USA).

### Quantification of frequency of prophage excision

The frequency of prophage excision under different conditions was quantified by quantitative PCR (qPCR). The number of chromosomes that are devoid of rac or other prophages was quantified using qPCR primers (Table S2) as previously described^7^. The number of total chromosomes was quantified by a reference gene, adenylosuccinate synthase (*purA*). A quantification method based on the relative amount of a target gene versus a reference gene was used^7^,^60^. Total DNA was isolated using a TIANamp Bacteria DNA kit (Tiangen, China) and was used as the template for the qPCR reaction using the Thermo Scientific Maxima SYBR Green/ROX qPCR Master Mix (Thermo scientific, Rockford, USA). The reaction and analysis was performed using the StepOne Real-Time PCR System (Applied Biosystems).

### Quantitative real-time reverse-transcription PCR (qRT-PCR)

For conducting qRT-PCR, total RNA was purified using a RNAprep Pure Cell/Bacteria kit (Tiangen, China). A total of 1 μg RNA was used for reverse transcription using the Reverse Transcription system kit (Promega, Madison, USA). The housekeeping gene *rrsG* (16S rRNA gene) was used to normalize the gene expression data. The remaining procedures followed those described above for the qPCR assay.

### Metabolic assay

Metabolic activities in the presence of carbenicillin were measured using the Biolog kit (Hayward, CA, USA) as previously described^26^. Briefly, overnight cultures of BW25113 and the isogenic deletion strains were diluted in LB medium to an OD_600_ of 0.05 and allowed to grow until the OD_600_ reached ~1.0. The cells were diluted to an OD_600_ 0.07 in 1 × IF-10a (Cat. No. 72264) and were further diluted 200-fold into a solution containing 1 × IF-10a, 1 × BioLog Redox Dye D (Cat. No. 74224), and 1 × Rich medium (2.0 g/L tryptone, 1.0 g/L yeast extract and 1.0 g/L NaCl) to a final OD_600_ of 0.0003. A volume of 100 μL of this cell suspension containing of 4 μg/mL carbenicillin was transferred into 96-well microtiter plates and was incubated at 37 °C without shaking. The metabolic activity was measured via the absorbance at 590 nm, which indicates the intracellular reduced state due to formazan (purple) formed from a tetrazolium dye.

## Additional Information

**How to cite this article**: Liu, X. *et al.* Physiological Function of Rac Prophage During Biofilm Formation and Regulation of Rac Excision in *Escherichia coli* K-12. *Sci. Rep.* **5**, 16074; doi: 10.1038/srep16074 (2015).

## Supplementary Material

Supplementary Information

## Figures and Tables

**Table 1:** Bacterial strains and plasmids used in this study.

Bacterial strains/Plasmids	Description	Source
BW25113	*lacI*^q^ *rrnB*_T14_ Δ*lacZ*_WJ16_ *hsdR*514 Δ*araBAD*_AH33_ Δ*rhaBAD*_LD78_	54
BL21(DE3)	F^*-*^*ompT hsdS*_*B*_*(r*_*B*_^*-*^*m*_*B*_^*-*^*) gal dcm λ*(DE3) Ω P_tacUV5_::T7 polymerase	Novagen
Δ*xisR*	*xisR* (*ydaQ*) deletion mutant derived from BW25113, Km^R^	54
Δ*ttcA*	*ttcA* (*ydaO*) deletion mutant derived from BW25113, Km^R^	54
Δ*hns*	*hns* deletion mutant derived from BW25113, Km^R^	54
Δ*rpoS*	*rpoS* deletion mutant derived from BW25113, Km^R^	54
Δ9	Δrac ΔCP4-57 ΔCPS-53 ΔDLP12 ΔQin Δe14 ΔCP4-6 ΔCPZ-55 ΔCP4-44 ΔKm^R^ derived from BW25113	7
ΔDLP12	Prophage DLP12 deletion mutant derived from BW25113	7
Δrac	Prophage rac deletion mutant derived from BW25113	7
ΔCP4-44	Prophage CP4-44 deletion mutant derived from BW25113	7
ΔCPZ-55	Prophage CPZ-55 deletion mutant derived from BW25113	7
ΔCP4-57	Prophage CP4-57 deletion mutant derived from BW25113	7
ΔQin	Prophage Qin deletion mutant derived from BW25113	7
ΔCP4-6	Prophage CP4-6 deletion mutant derived from BW25113	7
Δe14	Prophage e14 deletion mutant derived from BW25113	7
ΔCPS-53	Prophage CPS-53 deletion mutant derived from BW25113	7
Plasmids		
pCA24N	Cm^R^; *lacI*^q^, IPTG inducible expression vector in *E. coli*	55
pLP170	Car^R^, vector used for cell lysis assay in BW25113	59
pCA24N-*xisR*	Cm^R^; *lacI*^q^, vector for expressing *xisR* in *E. coli* host	This study
pCA24N-*ttcA*	Cm^R^; *lacI*^q^, vector for expressing *ttcA* in *E. coli* host	This study
pCA24N-*ttcA’*	Cm^R^; *lacI*^q^, vector for expressing the *ttcA* variant in *E. coli* host	This study
pET28b	Km^R^, expression vector	Novagen
pET28b-*xisR*	Km^R^, *lacI*^q^, pET28b P_*T7-lac*_:: *xisR* with six histidines tagged at the N-terminus	This study

Note: Km^R^, Cm^R^ and Car^R^ indicate kanamycin, chloramphenicol and carbenicillin resistance, respectively.
