# Supplementary material for: Physiological Function of Rac Prophage During Biofilm Formation and Regulation of Rac Excision in Escherichia coli K-12
Source: Sci Rep. 2015 Nov 4;5:16074. doi: 10.1038/srep16074 (PMC4632033; doi:10.1038/srep16074)
Supplement: Supplementary Information [file srep16074-s1.pdf]

Supplementary File

Physiological Function of Rac Prophage During Biofilm Formation and Regulation of Rac Excision in *Escherichia coli* K-12

Xiaoxiao Liu[1,+], Yangmei Li[1,2,+] , Yunxue Guo[1], Zhenshun Zeng[1,2], Baiyuan Li[1,2], Thomas K. Wood[3,4], Xingsheng Cai[1], Xiaoxue Wang[1,*]

[1]Key Laboratory of Tropical Marine Bio-resources and Ecology, Guangdong Key Laboratory of Marine Materia Medica, RNAM Center for Marine Microbiology, South China Sea Institute of Oceanology, Chinese Academy of Sciences, Guangzhou 510301, PR China,

[2]University of Chinese Academy of Sciences, Beijing 100049, China,

[3]Department of Chemical Engineering, [4]Department of Biochemistry and Molecular Biology, Pennsylvania State University, University Park, Pennsylvania 16802-4400

[*]Correspondence and requests for materials should be addressed to xxwang@scsio.ac.cn

[+]These authors contributed equally to this work.

Table S1. Mass spectrometry results of the purified YdaQ protein used in Fig. 7D. Peptide fragments observed were highlighted in different colors, and their loci in YdaQ protein were also shown.

Matched Protein ID：gi|90111254|ref|NP_415862.4| Description： Rac prophage; conserved protein [*Escherichia coli* str. K-12 substr. MG1655] >gi|90111254|
Amino acid (aa) sequence: MAQVIFNEEWMVEYGLMLRTGLGARQIEAYRQNCWVEGFHFKRVSPLGKPDSKRGIIWYNYPKINQFIKDS

Num	Query_ID	Pep_exp_mz	Pep_exp_mr	Pep_calc_mr	Pep_ms_delta	Start position	end position	Miss	Icon_ score	E-value	aa before	Peptide	aa after	Retention_time
1	5666	395.23	1182.67	1182.67	-0.0046	44	54	1	56.5	8.95E-06	R	VSPLGKPDSKR	G	6.4
2	3015	482.76	963.5	963.5	-0.0048	64	71	1	27.27	0.016312452	K	INQFIKDS	-	18.87
3	5153	577.3	1152.59	1152.6	-0.003	55	63	0	43.86	0.000265192	R	GIIWYNYPK	I	42.9
4	1728	390.2	778.39	778.4	-0.0053	26	31	0	39.46	0.001239978	R	QIEAYR	Q	7.93
5	7414	437.24	1308.7	1308.7	-0.0011	54	63	1	39.47	0.000581845	K	RGIIWYNYPK	I	23.77
6	10576	402.69	1606.75	1606.75	-0.0006	32	43	1	69.84	6.90E-07	R	QNCWVEGFHFKR	V	22.7
7	12591	754.04	2259.09	2259.07	0.0179	2	19	0	34.86	0.002057503	M	AQVIFNEEWMVEYGLMLR	T	41.58
8	1614	381.73	761.44	761.44	-0.0049	64	69	0	45.25	0.000213455	K	INQFIK	D	13.12
9	11714	633.02	1896.03	1896.03	0.0016	55	69	1	45.86	7.91E-05	R	GIIWYNYPKINQFIK	D	35.98
10	7689	445.91	1334.7	1334.69	0.0051	20	31	1	23.9	0.030960901	R	TGLGARQIEAYR	Q	14.1
11	8827	484.88	1451.63	1451.63	-0.0016	32	42	0	36.14	0.001058009	R	QNCWVEGFHFK	R	29.48
12	3713	514.29	1026.56	1026.57	-0.0066	44	53	0	50.76	4.78E-05	R	VSPLGKPDSK	R	6.95
13	1608	381.69	761.37	761.37	-0.0036	26	31	0	27.43	0.019607839	R	QIEAYR	Q	15.98
14	10582	402.94	1607.73	1607.73	-0.0033	32	43	1	53.34	2.76E-05	R	QNCWVEGFHFKR	V	24.57

Table S2. Nucleotide sequences of primers used in this study. Restriction enzyme sites are underlined. F indicates forward primer and R indicates reverse primer. Primers used in qPCR used in this study are the same as previously described[1].

**Primer Name/Purpose**	**Primer Sequence (listed 5' to 3')**
**DNA cloning and sequencing**	
pCA24N-*ttcA*-F	GCCCAAGAAAATCAACAAATTACAAAGAAAG
pCA24N-*ttcA*-R	CCTTTCACTTCAACCACATTCAGC
pCA24N-*ttcA*'-F	CCCTCGCAAAATCAAGAAATTAGTAAGAAAG
pCA24N-F	GATAACAATTTCACACAGAATT
pCA24N-R	GTCAGAGGTTTTCACCGTCATCA
pET28b-*xisR*-F	CTAG<u>CCATGG</u>GCAGCAGCCATCATCATCATCATCACGCACAAGTAATCTTTAATGAAG
pET28b-*xisR*-R	CCC<u>AAGCTT</u>TCATGAGTCTTTGATAAACTGATT
pET28b-*hns*-F	CTAG<u>CCATGG</u>GCATGAGCGAAGCACTTAAAATTCTGAA
pET28b-*hns*-R	CCC<u>AAGCTT</u>TTAGTGGTGGTGGTGGTGGTGTTGCTTGATCAGGAAATCGTC
T7-F	TAATACGACTCACTATAGGG
T7-R	TATGCTAGTTATTGCTCAG
**Amplify fragments for EMSA**	
rac attR-F	TTTGGAATTTTTTTCGGTGG
rac attR-R	AGATTTCTTATGCTGGGCG
rac attM-F	CTATATGCCTTTGATGCGGAG
rac attM-R	GTAAGGGCAAAAATCACAACTC
**qPCR**	
e14-F	GTGCAAACATCGGTGACGAA
e14-R	TTCAGCAGCTTAGCGCCTTC
rac-F	CTCCAGCATGGTATAGCTGTCTTTAC

rac-R	CAGATTTCTTATGCTGGGCGTTCCG
CP4-6-F	GCATCGCCCGGTTAGTTTTA
CP4-6-R	CACCTGCACTGCCTGATGTC
CPS-53-F	CGTACTTACCCCGCACTCCA
CPS-53 -R	GGCAGTGGCCAAAAATTGAA
DLP12-F	CAAAAGCCATTGACTCAGCAAGG
DLP12-R	ACGGATAAGACGGGCATAAATGA
CPZ-55-F	AGCACATCCCCCGAACG
CPZ-55-R	TTGACGAAGTGATTGTCCGC
CP4-57- F	AAGCATGTAGTACCGAGGATGTAGG
CP4-57-R	TATGTCTCCTCACCGTCTGGTCGG
purA-F	GGGCCTGCTTATGAAGATAAAGT
purA-R	TCAACCACCATAGAAGTCAGGAT
**qRT-PCR**	
xisR qPCR-F	TGGTTGAATACGGCCTGATG
xisR qPCR-R	GTACCAGATAATCCCTCGTTTGCT
rrsG-qPCR-F	TATTGCACAATGGGCGCAAG
rrsG-qPCR-R	ACTTAACAAACCGCTGCGT

Reference

1. Wang, X. *et al*. Cryptic prophages help bacteria cope with adverse environments. *Nat. Commun.* **1**, 147 (2010).
